# Parabrachial oxytocin receptor-expressing neurons link social observation of distress to defensive behavior

**DOI:** 10.64898/2026.08.06.743398

**Published:** 2026-08-07

**Authors:** Elena N. Judd, Jordan L. Pauli, Sayaka J. Kenmochi, Michael R. Bruchas, Richard D. Palmiter

## Abstract

The ability to detect and respond to threat signals in the environment, including those conveyed by the distress of a familiar social partner, is fundamental to survival and disrupted in a range of neuropsychiatric conditions. This study identifies oxytocin receptor (Oxtr)-expressing neurons within the lateral parabrachial nucleus (lPBN) of mice as a key node in the neural circuitry underlying threat-related and social behaviors. These Oxtr neurons are activated by aversive stimuli and by observing demonstrator mice in stressful situations, including foot shock or inflammatory pain. Chemogenetic inhibition of these neurons alters social proximity and pain contagion in observers without affecting general anxiety-like behavior. Inhibition also transiently suppresses non-social central sensitization. Direct activation of lPBN ^Oxtr^ neurons with a selective Oxtr agonist is anxiogenic and results in increased tactile sensitivity. Together, these findings suggest that lPBN ^Oxtr^ neurons are poised to integrate information about environmental threat, whether experienced directly or witnessed in a conspecific to coordinate appropriate defensive behavioral responses.

## Full Text Availability

The license terms selected by the author(s) for this preprint version do not permit archiving in PMC. The full text is available from the preprint server.

